# The Interaction Between Sour Jujube Kernel Peptide and Pea Starch and Its Effects on Starch Properties and In Vitro Digestibility

**DOI:** 10.3390/molecules31101718

**Published:** 2026-05-19

**Authors:** Chen Li, Wendi Zhu, Yunpo Huang

**Affiliations:** 1 College of Food and Health, Jinzhou Medical University, Jinzhou 121001, China; lic1@jzmu.edu.cn (C.L.); zhuwd@stu.jzmu.edu.cn (W.Z.); 2 Liaoning Provincial Professional Technology Innovation Center of Meat Processing and Quality—Safety Control, Jinzhou 121001, China

**Keywords:** sour jujube kernel peptide, pea starch, intermolecular interaction, in vitro starch digestibility, antioxidant

## Abstract

In this study, we systematically investigated the concentration-dependent effects of peptides derived from sour jujube kernel peptide (SJKP) on the multiscale structure, physicochemical properties, in vitro digestibility, and antioxidant activity of a complex formed between pea starch (PS) and SJKP. At an optimal SJKP content of 7.5% (*w*/*w*, based on starch dry basis), the slowly digestible starch (SDS) and resistant starch (RS) increased by 23.00% and 49.80%, respectively. X-ray diffraction (XRD) and Fourier-transform infrared spectroscopy (FT–IR) verified the formation of complexes and enhanced the short-range structural order of starch. Thermal analysis showed that the gelatinization enthalpy increased to 11.73 J/g, accompanied by an elevated gelatinization temperature and improved thermal stability. Conversely, at 15% SJKP content, RDS rebounded to 58.3% due to phase separation and structural collapse of the starch matrix. Intermolecular force analysis revealed that hydrogen bonding dominated at SJKP concentrations ≤ 7.5%, while hydrophobic interactions prevailed at concentrations ≥ 10%. SJKP incorporation also endowed the complexes with antioxidant capacity. These findings illustrate that SJKP interacts with pea starch via non-covalent bonds, forming a mixed gel network. Moderate SJKP levels can effectively modulate starch digestibility and functionality via regulating intermolecular interactions and multi-scale structure, offering promising potential for developing low-glycemic index (LGI) functional foods, including baked snacks, nutritional beverages, and diabetic-specific staple foods.

## 1. Introduction

Starch is the primary dietary carbohydrate source for humans, and its digestibility is closely associated with postprandial glycemic homeostasis, sustained energy release, and intestinal health [1]. Specifically, resistant starch (RS) can escape digestion in the small intestine and be fermented by gut microbiota in the colon, exerting prebiotic effects and regulating host metabolic health [2,3]. Pea starch, a plant-derived starch with high amylose content, has attracted widespread attention in the food industry due to its excellent gelatinization properties, wide source, and low cost [4,5]. However, native pea starch has a high proportion of rapidly digestible starch (RDS), which limits its application in low-glycemic index (LGI) and slow-energy-release functional foods [6].

In recent years, composite modification of starch using bioactive food components (including proteins, peptides, polysaccharides, and polyphenols) has become a mainstream strategy to tailor starch’s physicochemical properties and nutritional functions. Among these, plant-derived bioactive peptides have dual advantages. They can regulate starch digestion via multiple mechanisms (including inhibiting digestive enzyme activity, interacting with starch molecules to form ordered structures, and acting as physical barriers to enzymatic hydrolysis), while endowing starch-based foods with additional biological activities (such as antioxidant, hypoglycemic, and anti-inflammatory effects) [7,8]. Existing studies have confirmed that rice bran peptides [9], soy protein hydrolysates [10] and wheat germ peptides [11] can interact with starch via non-covalent bonds, thereby altering starch’s multi-scale structure and digestibility. However, most studies to date have focused on the linear influence of peptides on the physical properties of starch, so concentration-dependent nonlinear regulatory mechanisms and the underlying relationships between structure and function have not yet been sufficiently elucidated.

Ziziphus jujuba var. spinosa (sour jujube kernel) has been used in China since ancient times for both medicinal and culinary purposes and is rich in bioactive compounds [12]. Sour jujube kernel peptide (SJKP), prepared via enzymatic hydrolysis of sour jujube kernel protein, has been proven to have significant α-glucosidase inhibitory activity and free radical scavenging capacity, showing great potential in regulating glucose and lipid metabolism [13]. Compared with other cereal and legume protein peptides, SJKP has a unique amino acid profile with high content of glycine, proline, and arginine, which provides abundant sites for non-covalent interactions with starch molecules. Meanwhile, the dual biological activities of SJKP make it an ideal functional modifier for starch, which can simultaneously realize the regulation of starch digestibility and the improvement of antioxidant activity of starch-based foods [14,15]. However, to date, the mechanism of intermolecular interactions between SJKP and pea starch remains unclear.

Therefore, this study aims to investigate the interaction between SJKP and pea starch, characterize the multi-scale structural changes in the complexes, and elucidate the mechanisms underlying altered starch digestibility. The findings will provide a theoretical foundation and technical support for designing novel starch-based functional foods with slow-energy-release and antioxidant properties, while also promoting the valorization of pea and sour jujube seed resources.

## 2. Results and Discussion

### 2.1. Amino Acid Profile

The amino acid composition of SJKP is shown in Table 1. SJKP contains all essential amino acids (EAAs), with a total EAA content of 17.22 g per 100 g of protein. Leucine was the most abundant essential amino acid (3.30 g per 100 g of protein), while tryptophan was the limiting amino acid (AAS = 6.67%). For non-essential amino acids (NEAA), glycine was the most abundant (28.53 g/100 g protein), followed by proline (18.37 g/100 g protein), glutamic acid (9.48 g/100 g protein), and alanine (9.47 g/100 g protein). The total hydrophobic amino acid content (including glycine, alanine, valine, leucine, isoleucine, proline, and phenylalanine) was 66.39 g/100 g protein, providing a structural basis for hydrophobic interactions with starch. Meanwhile, SJKP contained 5.20 g/100 g protein aspartic acid and 7.88 g/100 g protein arginine, which are charged amino acids that can participate in electrostatic interactions and hydrogen bonding.

The unique amino acid composition of SJKP determines its ability to interact with starch via various non-covalent bonds. The hydroxyl and amino groups, which are abundant in polar amino acids, can form hydrogen bonds with the hydroxyl groups of starch molecules. Hydrophobic amino acids can penetrate the hydrophobic cavities of the amylose helix and form complexes within them via hydrophobic interactions. Furthermore, charged amino acids can form weak electrostatic interactions with the starch [16,17]. It is precisely these interactions that form the crucial basis for SJKP’s ability to regulate the structure and properties of pea starch.

### 2.2. Effect of SJKP on the Microstructure of Pea Starch

**Figure 1:**
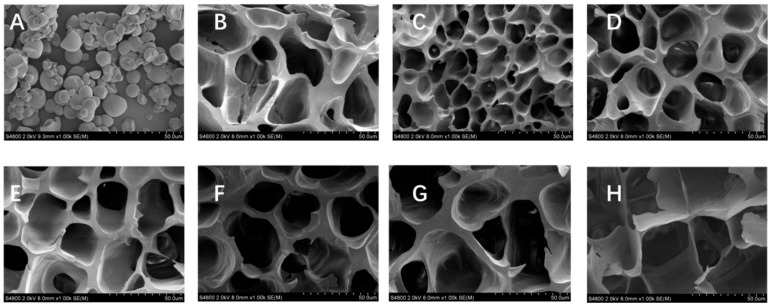
Microstructure of different samples. Native PS (**A**), gelatinized PS (**B**), gelatinized PS + 2.5% SJKP (**C**), PS + 5% SJKP (**D**), PS + 7.5% SJKP (**E**), PS + 10% SJKP (**F**), PS + 12.5% SJKP (**G**), and PS + 15% SJKP (**H**).

The microstructure of native PS, gelatinized PS, and PS–SJKP complexes is shown in Figure 1. Native pea starch exhibited intact, smooth, elliptical, or spherical granules with uniform particle size (Figure 1A). After gelatinization at 95 °C for 30 min, the granular structure of starch was completely destroyed, the crystalline regions were melted, and the sample showed a fragmented, rough, and porous amorphous structure with no continuous gel network (Figure 1B).

After the addition of SJKP, the microstructure of the complexes showed a significant concentration-dependent change. At low SJKP concentrations (2.5–7.5%), the complexes formed a denser and more continuous three-dimensional gel network compared with the gelatinized pure PS (Figure 1C–E). This suggests the involvement of hydrogen bonding, as further supported by the FT–IR and intermolecular force results presented in Section 2.3.2 and Section 2.6, which promotes the orderly arrangement of starch double helices, inhibits the fragmentation of starch molecules during gelatinization, and thus forms a dense and stable gel network [18,19].

When the SJKP concentration increased to 10–15%, the gel network structure gradually deteriorated (Figure 1F–H). The 10% SJKP group (Figure 1F) showed a slight thinning of the pore walls, while the 12.5% SJKP group (Figure 1G) exhibited obvious pore wall rupture and structural discontinuity. The 15% SJKP group (Figure 1H) further evolved into a lamellar and striped morphology, with complete loss of the three-dimensional network structure and significant reduction in structural order. This is because at high concentrations, excessive SJKP molecules induce phase separation via hydrophobic interactions, which weaken the intermolecular hydrogen bonds between starch molecules, destroy the continuity of the gel network, and eventually lead to structural dissociation and collapse [20,21].

### 2.3. Effect of SJKP on the Multi–Scale Structure of Pea Starch

#### 2.3.1. Long–Range Ordered Structure (XRD Analysis)

**Figure 2:**
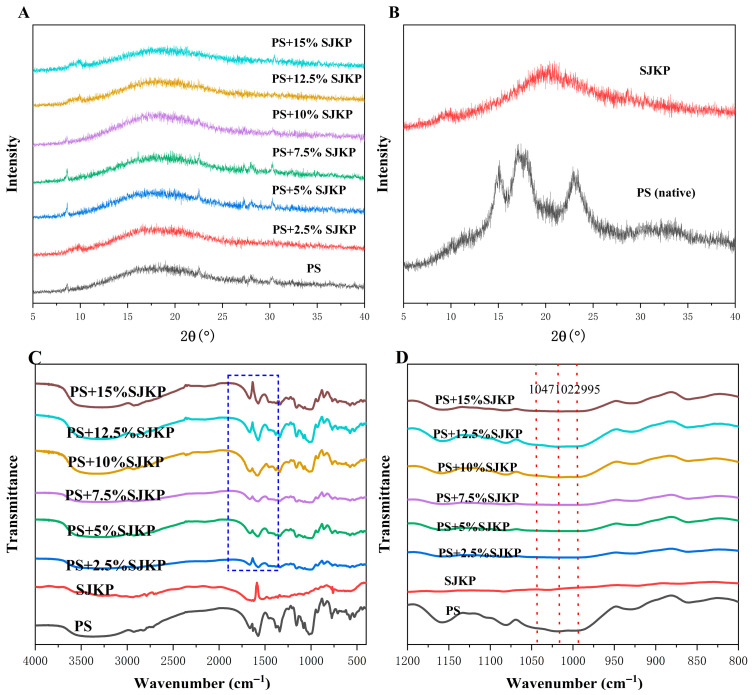
The XRD patterns of native PS, gelatinized PS, and PS–SJKP complexes (**A**) and zoom-in of the 15–25° 2θ region (**B**), and FT–IR spectra (**C**,**D**) of different concentrations of PS–SJKP.

The XRD patterns are shown in Figure 2A (full patterns) and Figure 2B (zoom-in of the 15–25° 2θ region). Native pea starch displayed a typical C-type crystalline structure, with characteristic diffraction peaks at 15.0°, 17.0°, 18.0°, 20.0°, and 23.0° (2θ), which is consistent with previous reports on pea starch [22]. After gelation, all of the distinct crystalline peaks observed in the original starch component disappeared, and only a broad, diffuse “amorphous region” was observed between 15° and 25° (2θ). This suggests that the crystalline structure of the starch was completely destroyed during the gelation process, causing the sample to transition to an amorphous state [23].

Under the experimental conditions (95 °C, 30 min), the SJKP–PS complex was completely gelatinized, as evidenced by the loss of its characteristic C-type diffraction peaks. In this fully amorphous state, the addition of SJKP does not induce immediate recrystallization during the short preparation period. The observed functional changes are therefore attributed to molecular interactions in the amorphous matrix rather than to the formation of a new crystalline phase.

#### 2.3.2. Short-Range-Ordered Structure (FT–IR Analysis)

The FT–IR spectra of the PS–SJKP complex are shown in Figure 2C,D. The absorption peaks characteristic of starch were observed in the spectra of all samples. The broad peak at 3400 cm^−1^ corresponds to the stretching vibration of the O-H group, the peak at 2930 cm^−1^ to the stretching vibration of the C-H group, and the peaks at 1150 cm^−1^, 1080 cm^−1^, and 1022 cm^−1^ correspond to the stretching vibrations of the C-O and C-C groups within the starch molecule. With the increase in SJKP concentration, the characteristic absorption peaks of amide I (1650 cm^−1^) and amide II (1540 cm^−1^) of the peptide gradually intensified, but no new characteristic absorption peaks appeared in the spectra of the complexes [24].

The absorption intensity ratio at 1047/1022 cm^−1^ (R1047/1022) is frequently used to assess the short–range order structure of starch; a higher ratio indicates a higher degree of molecular order in the starch [25]. While the R1047/1022 value for gelled pure PS was 0.682 ± 0.015, the value for the complex initially increased with increasing SJKP concentration, but then decreased again, reaching a maximum value of 0.895 ± 0.021 at 7.5% SJKP (Table S1). This indicates that low concentration of SJKP significantly enhances the short–range ordered structure of starch. The ratio of 995/1022 cm^−1^ (R995/1022) is related to the formation of hydrogen bonds between starch molecules. The R995/1022 value of the complexes also showed the same trend as R1047/1022, confirming that hydrogen bonding plays a key role in the formation of ordered structure at low SJKP concentrations.

When the SJKP concentration exceeded 10%, the R1047/1022 and R995/1022 values decreased significantly, indicating that the short-range-ordered structure of starch was destroyed, which is consistent with the XRD results showing the disappearance of crystalline peaks and the SEM results showing structural collapse.

#### 2.3.3. Helical Structure (Iodine Binding Capacity Analysis)

**Figure 3:**
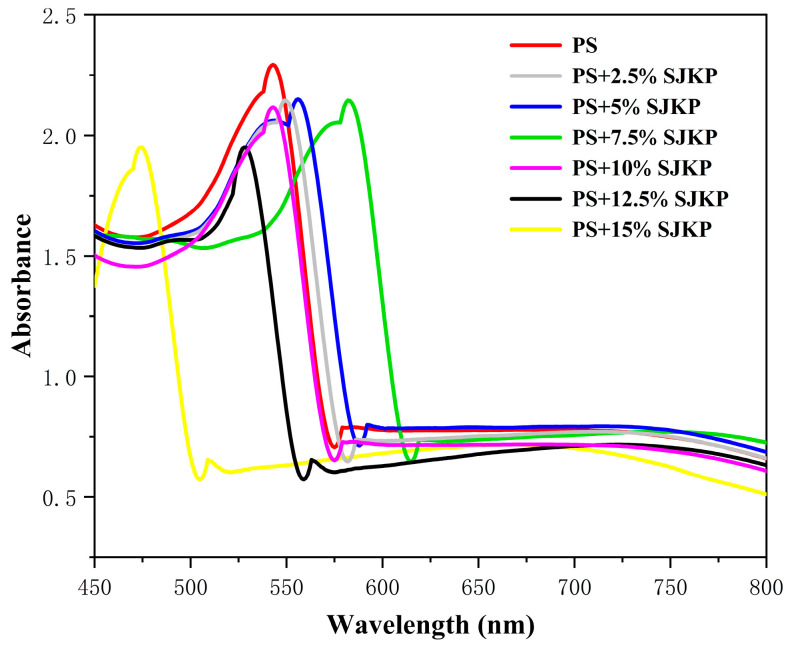
The iodine binding capacity of different samples.

Iodine can enter the hydrophobic cavity of amylose single helices to form a blue complex, and the maximum absorption wavelength (λmax) and absorbance of the complex are directly related to the length and integrity of amylose helices [26]. Therefore, iodine-binding capacity analysis was performed to evaluate the effect of SJKP on the helical structure of amylose, and the results are shown in Figure 3.

Compared with gelatinized pure PS, all PS–SJKP complexes showed a significant decrease in iodine binding absorbance, indicating that SJKP competitively occupies the binding sites of iodine in amylose helices, reduces the number of available binding sites for iodine, and thus inhibits the formation of starch–iodine complexes [27]. The reduced iodine-binding absorbance suggests that SJKP competitively occupies or physically blocks the iodine-binding sites on amylose helices. However, this does not definitively prove intra-helical inclusion, as surface adsorption or physical entanglement could also contribute. Direct structural evidence (e.g., NMR or molecular dynamics simulation) is needed to clarify the exact binding mode.

The λmax of the complexes showed a biphasic change with the increase in SJKP concentration: at low concentrations (≤7.5%), λmax showed a red shift from 586 nm (pure PS) to 598 nm (7.5% SJKP). This red shift may suggest a more extended or ordered helical conformation, but direct structural evidence (e.g., circular dichroism or NMR) is needed to confirm this hypothesis. Similar red shifts have been observed in amylose-lipid complexes. At high concentrations (≥10%), λmax showed a significant blue shift, decreasing to 572 nm at 15% SJKP, indicating that excessive SJKP disrupts the integrity of amylose helices, leads to the unwinding and shortening of the helical structure, and thus causes a blue shift in λmax.

The iodine binding capacity results clearly reveal the dual effect of SJKP on the amylose helical structure: at low concentrations, SJKP acts as a structural inducer to promote the formation of stable and elongated single helices; at high concentrations, excessive SJKP disrupts the helical structure, leading to the loss of ordered structure.

### 2.4. Effect of SJKP on the Physicochemical Properties of Pea Starch

#### 2.4.1. Pasting Properties

**Figure 4:**
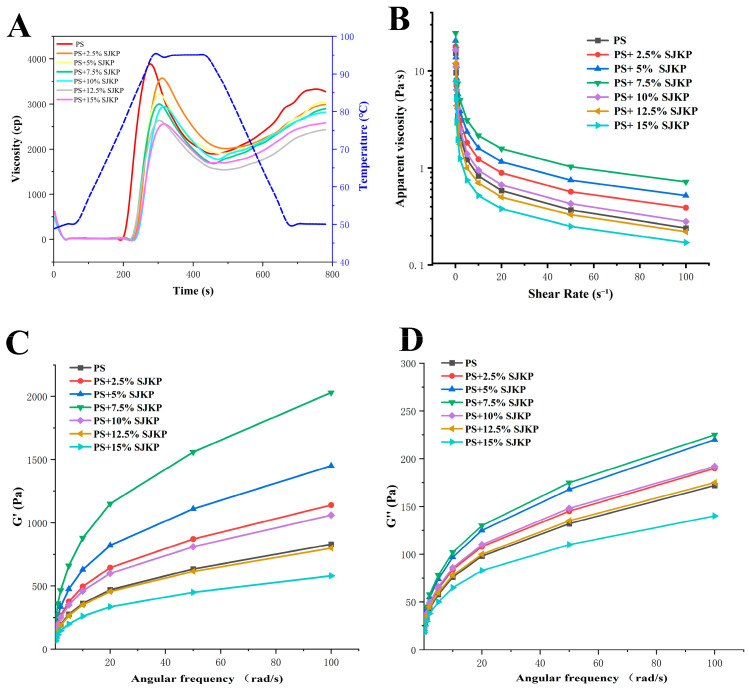
The pasting properties (**A**), the apparent viscosity (**B**), G′ (**C**), and G″ (**D**) of different samples.

The pasting properties of PS–SJKP complexes are shown in Table 2 and Figure 4A. With the increase in SJKP concentration, the peak viscosity (PV), trough viscosity (TV), and final viscosity (FV) of the complexes decreased significantly (*p* < 0.05). This is because SJKP inhibits the swelling and gelatinization of starch particles during heating by competitively binding to starch and water molecules, thereby reducing the viscosity of the entire system. Meanwhile, the decrease in starch content in the mixed system also contributes to the decrease in viscosity.

Breakdown (BD) reflects the thermal shear stability of starch granules during gelatinization, with a lower BD indicating higher stability. The BD value of the complexes decreased significantly from 2111.40 cP (pure PS) to 902.00 cP (15% SJKP) with the increase in SJKP concentration, indicating that SJKP effectively inhibits the rupture of starch granules during heating, enhances the thermal shear stability of starch, and reduces the leaching of amylose. This is because SJKP can adsorb on the surface of starch granules via hydrogen bonding, form a protective film on the granule surface, limit the penetration of water into the granules, and thus maintain the structural integrity of starch granules during heating [28].

Setback (SB) reflects the short-term retrogradation tendency of starch during cooling, with a higher SB indicating a stronger retrogradation tendency. The SB value of the complexes decreased significantly from 1388.60 cP (pure PS) to 912.24 cP (15% SJKP) (*p* < 0.05), indicating that SJKP can effectively inhibit the short-term retrogradation of pea starch. This is because SJKP can form hydrogen bonds with amylose molecules, hinder the re–association and re-crystallization of amylose molecules during cooling, and thus inhibit retrogradation [29].

The pasting temperature (PT) and peak time (Pt) tended to increase with SJKP addition, indicating a delay in gelatinization. However, the changes were not strictly linear: for example, PT fluctuated between 84.04 °C and 84.98 °C without a clear concentration-dependent pattern (Table 2). Therefore, we conclude that SJKP generally delays gelatinization, but the effect is not strictly proportional to concentration.

#### 2.4.2. Rheological Properties

The flow curves of PS–SJKP complexes are shown in Figure 4B. All samples exhibited typical shear-thinning behavior, with apparent viscosity decreasing as shear rate increased, which is characteristic of non-Newtonian plastic fluids. This is because the three-dimensional network structure of the starch paste is disrupted by shear forces, leading to a decrease in viscosity. At low SJKP concentrations (≤7.5%), the apparent viscosity of the complexes was significantly higher than that of pure PS, with the 7.5% SJKP group having the highest viscosity. At high SJKP concentrations (≥10%), the apparent viscosity decreased sharply, reaching its lowest at 15% SJKP. This is due to the phase separation and structural collapse induced by hydrophobic interactions, which destroys the continuity of the gel network, leading to a significant decrease in viscosity [30].

The results of frequency sweep tests are shown in Figure 4C,D. For all samples, the storage modulus (G′) and loss modulus (G″) increased with the increase in angular frequency, and G′ was always higher than G″ in the whole frequency range, indicating that all samples exhibited typical weak-gel behavior. The G′ value of the complexes first increased and then decreased with the increase in SJKP concentration, reaching a maximum at 7.5% SJKP, indicating that the 7.5% SJKP group formed the most stable and elastic three-dimensional gel network. When the SJKP concentration exceeded 10%, G′ and G″ decreased significantly, confirming that the gel network structure was severely damaged at high concentrations, which is consistent with the microstructural and viscosity results.

The discrepancy between RVA pasting viscosity and rheometer-measured apparent viscosity may arise from differences in shear conditions (RVA uses a high initial shear of 960 rpm followed by 160 rpm, while the rheometer uses controlled shear rate ramping from 0.1 to 100 s^−1^), temperature profiles (RVA includes heating, holding, and cooling stages, whereas rheometer measurements were performed at constant 50 °C), and measurement principles (RVA measures resistance to a paddle, while the rheometer uses parallel plate geometry). Therefore, the two methods provide complementary rather than identical information. The higher RVA viscosity of pure PS reflects its greater granule swelling capacity under high-temperature shearing, whereas the lower apparent viscosity of pure PS under steady shear at 50 °C indicates a weaker gel network after cooling, consistent with its fragmented microstructure observed in SEM (Figure 1B).

#### 2.4.3. Thermal Properties

The thermal properties of PS–SJKP complexes are shown in Table 3. With the increase in SJKP concentration from 0% to 15%, the onset temperature (To), peak temperature (Tp), and conclusion temperature (Tc) of the complexes increased systematically: To rose from 61.33 °C to 66.95 °C, and Tp from 66.55 °C to 73.20 °C. This indicates that SJKP significantly enhances the thermal stability of pea starch, and higher temperature and energy input are required to destroy the ordered structure of starch and achieve gelatinization. This is because hydrogen bonds formed between SJKP and starch molecules enhance the intermolecular forces of the starch system, further increasing the structural stability of starch, thus increasing the gelatinization temperature.

The gelatinization temperature range (Tc–To) first narrowed and then widened with the increase in SJKP concentration, reaching the minimum of 11.56 °C at 7.5% SJKP, and the maximum of 13.13 °C at 15% SJKP. A narrower gelatinization temperature range indicates a higher uniformity of starch granule gelatinization. This result confirms that low concentration of SJKP improves the synchronization of starch granule gelatinization, while excessive SJKP leads to structural heterogeneity and disorder of the system, resulting in a wider gelatinization temperature range.

The gelatinization enthalpy (ΔH) reflects the energy required to destroy the ordered structure of starch, including double helices and crystalline regions. At low SJKP concentrations (≤7.5%), the increase in ΔH is consistent with enhanced hydrogen bonding and short–range order, as supported by FT–IR (R1047/1022) and the intermolecular force analysis (Table 4). At high concentrations (≥10%), the significant decrease in ΔH (from 11.73 J/g at 7.5% to 8.65 J/g at 15% SJKP) is attributed to phase separation and disruption of the starch gel network, as evidenced by SEM (Figure 1F–H) and the dominance of hydrophobic interactions (Table 4). The loss of structural integrity reduces the energy required for gelatinization, leading to a lower ΔH.

### 2.5. Effect of SJKP on the In Vitro Digestibility of Pea Starch

#### 2.5.1. Starch Fractions (RDS, SDS, RS)

**Figure 5:**
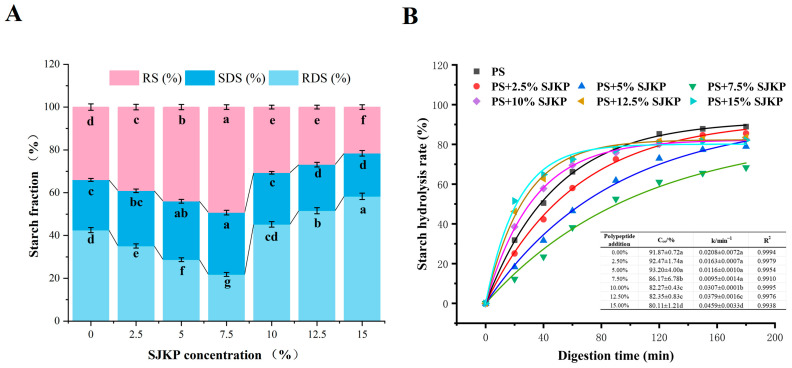
Starch fractions (RDS, SDS, and RS) of different samples (**A**) and simulated starch hydrolysis curves of PS–SJKP composites at different concentrations (**B**). The solid lines represent the fitted curves using the first–order kinetic model. Values with different lowercase letters are significantly different (*p* < 0.05).

The effect of SJKP on the RDS, SDS, and RS contents of pea starch is shown in Figure 5A. The RDS content of the complexes showed a typical V-shaped change with the increase in SJKP concentration, whereas the SDS and RS contents showed an inverted V-shaped change, which was completely opposite to that of RDS.

At low SJKP concentrations (≤7.5%), the RDS content decreased significantly (*p* < 0.05), from 42.5 ± 1.2% (pure PS) to the minimum of 21.8 ± 0.9% at 7.5% SJKP, a reduction of 48.7%. Meanwhile, the SDS and RS contents increased significantly, reaching the maximum at 7.5% SJKP, with SDS at 28.9 ± 0.8% (an increase of 23.0%) and RS at 48.7 ± 1.2% (an increase of 49.8%). Conversely, at high SJKP concentrations (≥10%), the RDS content increased sharply, reaching 58.3 ± 1.5% at 15% SJKP, which was significantly higher than that of pure PS (*p* < 0.01). Meanwhile, the SDS and RS contents decreased significantly to 20.1 ± 0.6% and 18.9 ± 0.7%, respectively, at 15% SJKP. This rebound in digestibility is mainly due to the phase separation and structural collapse of the starch matrix induced by excessive SJKP. The destruction of the gel network re-exposes the starch chains to digestive enzymes, providing abundant enzymatic hydrolysis sites, and thus significantly accelerating the digestion rate [31]. Although free SJKP at high concentrations has a certain inhibitory effect on enzyme activity, the dominant effect of structural collapse far outweighs the enzyme-inhibitory effect, eventually leading to a significant increase in RDS content.

#### 2.5.2. Digestion Kinetics

The in vitro starch hydrolysis curves and first-order kinetic model fitting results are shown in Figure 5B. The first-order kinetic model can well fit the digestion process of all samples (R^2^ > 0.99). The estimated maximum digestibility (C∞) of the complexes first decreased and then increased with the increase in SJKP concentration, reaching a minimum of 86.17 ± 6.78% at 7.5% SJKP, which was significantly lower than that of pure PS (91.87 ± 0.72%, *p* < 0.001). This indicates that the 7.5% SJKP group has the strongest resistance to enzymatic hydrolysis, with the lowest proportion of starch that can be ultimately digested. When the SJKP concentration exceeded 10%, C∞ increased again, reaching 80.11 ± 1.21% at 15% SJKP. It was confirmed that structural breakdown significantly increased the content of digestible starch.

The digestion rate constant (k) showed the opposite trend to C∞, with an initial decrease followed by an increase. The minimum k value of 0.0095 ± 0.0014 min^−1^ was observed at 7.5% SJKP, indicating the slowest digestion rate. In contrast, the highest k value of 0.0459 ± 0.0033 min^−1^ was found at 15% SJKP, reflecting the fastest digestion rate. These results are completely consistent with the changes in RDS, SDS, and RS content, and further confirm that a moderate SJKP concentration can effectively slow down the starch digestion rate, while excessive SJKP will accelerate digestion.

### 2.6. Intermolecular Interaction Forces Between SJKP and Pea Starch

The relative contributions of different intermolecular forces to the stability of PS–SJKP complexes were evaluated by measuring changes in final viscosity after treatment with specific chemical disruptors, and the results are shown in Table 4.

Treatment with 0.5 M NaCl (to disrupt ionic bonds) caused minimal changes in the final viscosity of all samples, with ΔFV values ranging from −7.50% to +5.20%, and no consistent concentration–dependent trend. This indicates that ionic interactions are not the dominant force stabilizing the PS–SJKP complexes. Although SJKP contains a certain amount of aspartic acid (acidic) and arginine (basic), which are charged amino acids, the pH of the reaction system (6.5–7.0) is close to the isoelectric point of most peptides, resulting in a low net charge of SJKP molecules. Meanwhile, the hydroxyl groups of starch molecules have very low charge density under neutral conditions. These two factors lead to the weak electrostatic interaction between SJKP and pea starch.

Treatment with 6 M urea (to disrupt hydrogen bonds) caused a significant decrease in the final viscosity of the complexes, and the effect showed a strong concentration dependence. At low SJKP concentrations (≤7.5%), the ΔFV values increased significantly with the increase in SJKP concentration, reaching −52.30% at 7.5% SJKP (*p* < 0.001). This indicates that hydrogen bonding is the dominant force stabilizing the complexes at low SJKP concentrations. The abundant polar amino acid residues in SJKP can form multiple hydrogen bonds with the hydroxyl groups of starch molecules, which promotes the cross-linking of starch molecules and the formation of a stable gel network. When hydrogen bonds are disrupted by urea, the network structure collapses, leading to a significant decrease in viscosity. At high SJKP concentrations (≥10%), the disruptive effect of urea gradually weakened, with ΔFV decreasing to −18.5% at 15% SJKP, indicating that the contribution of hydrogen bonds to the stability of the complexes is significantly reduced at high concentrations.

In contrast, treatment with 2% SDS (to disrupt hydrophobic interactions) showed the opposite trend. At low SJKP concentrations (≤7.5%), the ΔFV values were only between −8.3% and −12.5%, with no significant change compared with the control, indicating that hydrophobic interactions are not the dominant force at low concentrations. At high SJKP concentrations (≥10%), the disruptive effect of SDS was significantly enhanced, with ΔFV reaching −48.90% at 15% SJKP (*p* < 0.01), confirming that hydrophobic interactions become the dominant force stabilizing the system at high concentrations. Excessive SJKP molecules aggregate via hydrophobic interactions between hydrophobic amino acid residues (valine, leucine, isoleucine, proline, and alanine), leading to phase separation between the peptide and starch phases. Although the total content of valine and leucine in SJKP is 5.73 g/100 g protein, the total hydrophobic amino acid content is as high as 66.39 g/100 g protein, especially the high content of glycine, alanine, and proline, which can also participate in hydrophobic interactions. Meanwhile, the high concentration of SJKP leads to the accumulation of hydrophobic groups, which amplifies the effect of hydrophobic interactions, making it the dominant force in the system.

Combined with the structural characterization results, the intermolecular interaction mechanism between SJKP and pea starch is clearly elucidated: at SJKP concentrations ≤ 7.5%, hydrogen bonding is the dominant force, which promotes the formation of complexes and a dense gel network; at concentrations ≥ 10%, hydrophobic interactions become dominant, which induces phase separation and structural collapse of the starch matrix. This transition of dominant intermolecular forces is the core mechanism driving the non–linear changes in the structure and functional properties of the complexes.

### 2.7. Antioxidant Activity of PS–SJKP Complexes

**Figure 6:**
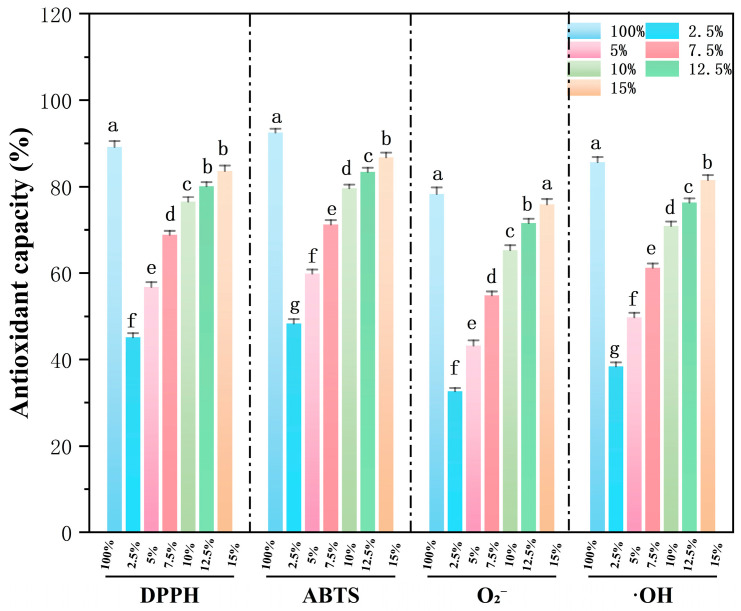
The antioxidant capacity of different samples. Values with different lowercase letters are significantly different (*p* < 0.05).

The antioxidant activity of PS–SJKP complexes is shown in Figure 6. The radical-scavenging activity of the complexes showed a distinct biphasic trend with the increase in SJKP concentration.

At low SJKP concentrations (≤7.5%), the radical scavenging activity of the complexes was significantly inhibited (*p* < 0.05). Compared with the pure SJKP solution with the same concentration, the DPPH scavenging rate of the 2.5% SJKP complex decreased from 78.30% to 48.50%, and the ABTS scavenging rate decreased from 85.6% to 48.5%. This reduction in antioxidant activity may be due to physical entrapment within the gel network, surface adsorption, or partial encapsulation. The definitive mechanism remains to be elucidated. The encapsulation effect hinders the contact between the active groups of SJKP and free radicals, thus inhibiting the expression of antioxidant activity [32,33]. The encapsulation efficiency of SJKP by starch helices reaches the maximum at 7.5% SJKP, resulting in the lowest antioxidant activity of the complex.

At high SJKP concentrations (≥10%), the radical-scavenging capacity of the complexes recovered sharply. The DPPH and ABTS scavenging rates of the 15% SJKP complex reached 85.2% and 81.4%, respectively, which showed no significant difference from the pure SJKP solution at the same concentration (*p* > 0.05). This is because the starch helical encapsulation capacity is saturated at high SJKP concentrations, and excessive SJKP molecules are dissociated from the starch matrix due to phase separation induced by hydrophobic interactions. The free SJKP molecules re-expose their active groups, which can fully react with free radicals, thus restoring antioxidant activity to the level of a pure SJKP solution.

The results of antioxidant activity reveal a unique “encapsulation–release” regulation mechanism of starch structure on the antioxidant activity of SJKP: at low concentrations, SJKP is encapsulated within starch helices, with low antioxidant activity but excellent digestion resistance; at high concentrations, SJKP is released from the starch matrix, with fully expressed antioxidant activity but accelerated starch digestion. This provides a new strategy for the design of functional foods with controlled release of both carbohydrates and antioxidants.

## 3. Materials and Methods

### 3.1. Materials and Reagents

Prior to complex preparation, the quality of sour jujube kernel peptide (SJKP) and pea starch (PS) was confirmed by our laboratory. SJKP (molecular weight distribution: 92.3% < 1000 Da, protein content: 89.2 ± 0.35%, dry basis) was supplied by Ronglin Biotechnology Co., Ltd. (Xi’an, China). The protein content was determined by the Kjeldahl method, and the molecular weight distribution by HPLC. Pea starch (PS, total starch content: 88.6 ± 0.42%, amylose content: 35.2 ± 0.28%, dry basis) was obtained from Zhongchen Biological Company (Zaozhuang, China). Total starch content was measured using a Megazyme kit (Megazyme Ltd., Bray, County Wicklow, Ireland), and amylose content by the iodine-binding colorimetric method. Porcine pancreatic α-amylase (P7545, 30 U/mg solid), amyloglucosidase (A7095, ≥300 U/mL), and glucose assay kit (GOPOD method, MAK013) were purchased from Sigma-Aldrich Co., Ltd. (St. Louis, MO, USA). Sodium dodecyl sulfate (SDS), urea, sodium chloride, and all other chemical reagents were of analytical grade, purchased from Shanghai Aladdin Biochemical Technology Co., Ltd. (Shanghai, China).

### 3.2. Amino Acid Composition Analysis of Sour Jujube Kernel Peptide

Amino acid composition of SJKP was determined according to the method of Li et al. (2024) using an Ultimate 3000 high-performance liquid chromatography (HPLC) system (Thermo Fisher Scientific Inc., Waltham, MA, USA) [34]. The amino acid score (AAS) was calculated following the FAO/WHO (2013) adult essential amino acid requirement pattern.

### 3.3. Preparation of Pea Starch–Sour Jujube Kernel Peptide Complex (PS–SJKP)

Pea starch (10.00 g, dry basis) was mixed with SJKP at mass ratios of 0, 0.25, 0.50, 0.75, 1.00, 1.25, and 1.50 g to achieve final SJKP contents of 0, 2.5%, 5.0%, 7.5%, 10.0%, 12.5%, and 15.0% (*w*/*w*, based on starch dry basis). Each mixture was dispersed in 50 mL of ultrapure water and stirred continuously at 500 rpm for 20 min at room temperature to ensure a uniform dispersion. The suspension was then heated in a water bath at 95 °C with continuous stirring for 30 min to achieve complete gelation (gelation degree > 98%, measured using the double-beam method). The resulting paste was immediately cooled to room temperature in an ice-water bath. For XRD, FT–IR, and amino acid analysis, the sample was freeze-dried at −50 °C for 48 h (SCIENTZ-10N, Ningbo Scientz Biotechnology Co., Ltd., Ningbo, China), then ground, sieved through a 100–mesh sieve, and stored in a desiccator at 4 °C until analysis.

### 3.4. Microstructural Analysis

The microstructure of freeze-dried PS–SJKP complexes was observed using a scanning electron microscope (SEM) (JEOL Ltd., Tokyo, Japan). Samples were fixed on aluminum stubs with conductive adhesive, sputter-coated with gold for 120 s under vacuum and then imaged at an accelerating voltage of 10 kV with magnifications of 1000×. At least 5 different fields of view were observed for each sample to ensure the representativeness of the results.

### 3.5. Multi–Scale Structural Characterizations

#### 3.5.1. X-Ray Diffraction (XRD) Analysis

XRD patterns of the samples were acquired using a D8 Advance X-ray diffractometer (Bruker Inc., Karlsruhe, Germany). The scanning range was 4° to 50° (2θ) with a step size of 0.02° and a scanning rate of 4°/min.

#### 3.5.2. Fourier-Transform Infrared Spectroscopy (FT–IR) Analysis

FT–IR spectra of the samples were recorded using a Nicolet iS50 Fourier-transform infrared spectrometer (Thermo Fisher Scientific Inc., Waltham, MA, USA). Freeze-dried samples were mixed with dry KBr at a mass ratio of 1:100, ground to a fine powder, and pressed into transparent pellets. The spectra were collected in the wavenumber range of 4000–400 cm^−1^ with 64 scans and a resolution of 4 cm^−1^. The absorbance intensity ratios of 1047/1022 cm^−1^ and 995/1022 cm^−1^ were calculated to evaluate the short-range ordered structure and hydrogen bonding changes in starch. All measurements were performed in triplicate.

#### 3.5.3. Iodine Binding Capacity Analysis

Iodine binding capacity was measured to evaluate the changes in amylose helical structure of starch after complexation with SJKP, according to the method of Kou & Gao (2019) [26]. Full-wavelength scanning was performed from 400 to 800 nm using a UV-2600 ultraviolet-visible spectrophotometer (Shimadzu Corporation, Kyoto, Japan), with ultrapure water as the blank. Blank samples containing corresponding concentrations of SJKP without starch were prepared to eliminate the background interference of SJKP.

### 3.6. Evaluation of Physicochemical Properties

#### 3.6.1. Pasting Properties Analysis

Pasting properties of the samples were evaluated using a Rapid Visco Analyzer (RVA 4500, Perten Instruments, Macquarie Park, NSW, Australia). Briefly, the total solid (starch + SJKP) was kept constant at 3.00 g, with the PS content varying accordingly. This ensures that any changes in pasting properties are not simply due to varying total solid content. The test procedure was set as follows: the sample was held at 50 °C for 1 min, heated from 50 °C to 95 °C at a rate of 12 °C/min, held at 95 °C for 2.5 min, then cooled from 95 °C to 50 °C at a rate of 12 °C/min, and finally held at 50 °C for 2 min. The stirring speed was 960 rpm for the first 10 s, then maintained at 160 rpm for the rest of the test. Parameters recorded included pasting temperature (PT), peak time (Pt), peak viscosity (PV), trough viscosity (TV), final viscosity (FV), breakdown (BD), and setback (SB), all expressed in centipoise (cP).

#### 3.6.2. Rheological Properties Analysis

Rheological properties of the samples were measured using a MCR 302 rheometer (Anton Paar GmbH, Graz, Austria) equipped with a 40 mm parallel plate geometry with a gap of 1000 μm. Freshly prepared starch pastes (after gelatinization at 95 °C for 30 min) were loaded onto the plate, and the excess sample was wiped off. Silicone oil was applied to the edge of the sample to prevent water evaporation during measurement. All measurements were performed at 50 °C after equilibration for 5 min.

Strain sweep test: performed in the strain range of 0.01–100% at a fixed angular frequency of 10 rad/s to determine the linear viscoelastic region (LVR) of the samples.

Flow curve test: performed in the shear rate range of 0.1–100 s^−1^ to record the apparent viscosity changes in the samples.

Frequency sweep test: performed in the angular frequency range of 0.1–100 rad/s within the LVR (strain = 0.5%), to record the changes in storage modulus (G′) and loss modulus (G″) of the samples.

#### 3.6.3. Thermal Properties Analysis

Thermal properties of the samples were analyzed using a DSC 8500 differential scanning calorimeter (PerkinElmer Inc., Waltham, MA, USA). Samples (3.0–5.0 mg, dry starch basis) were accurately weighed into an aluminum hermetic pan, and ultrapure water was added at a water/starch ratio of 3:1 (*v*/*w*). The pan was hermetically sealed and equilibrated at 4 °C for 24 h before measurement. An empty sealed aluminum pan was used as the reference. Scanning was performed from 25 °C to 100 °C at a heating rate of 10 °C/min, with a nitrogen flow rate of 20 mL/min. Onset temperature (To), peak temperature (Tp), conclusion temperature (Tc), and gelatinization enthalpy (ΔH, calculated based on dry starch mass) were recorded using Pyris software 13.3.1 (PerkinElmer Inc., 940 Winter Street, Waltham, MA, USA).

### 3.7. In Vitro Digestibility Analysis

In vitro starch digestibility was evaluated using a simulated gastrointestinal digestion system according to the Englyst method with minor modifications, and the effect of SJKP on the accuracy of the method was systematically evaluated.

Sample preparation: 200.00 mg of sample (dry starch basis) was weighed into a 50 mL centrifuge tube, and 15 mL of sodium acetate buffer (0.1 M, pH 5.2) was added. The mixture was vortexed for 1 min, and pre–incubated in a 37 °C water bath for 10 min.

Enzyme solution preparation: Porcine pancreatic α-amylase (2.0 g) was dissolved in 24 mL of sodium acetate buffer, vortexed for 10 min, and centrifuged at 3000× *g* for 10 min. 18 mL of the supernatant was mixed with 3 mL of amyloglucosidase (300 U/mL) to prepare the mixed enzyme solution, which was prepared fresh before each experiment.

Digestion procedure: 5 mL of the mixed enzyme solution was added to the sample tube, and the mixture was incubated in a 37 °C water bath with shaking at 150 rpm. Aliquots of 0.5 mL were taken at 0, 20, and 120 min of digestion, and immediately mixed with 4 mL of absolute ethanol to inactivate the enzyme. The glucose content in the supernatant was determined using the GOPOD method.

Calculation of starch fractions: Rapidly digestible starch (RDS), slowly digestible starch (SDS), and resistant starch (RS) were calculated using the following formulas:

(1) $$\mathrm{Hydrolysis}\;\mathrm{rate}\;\left(\%\right)=\frac{Gt\times 0.9\;}{\mathrm{starch}\;\mathrm{mass}\;(\mathrm{mg})\;}\times 100$$

(2) $$\mathrm{RDS}\;\left(\%\right)=\frac{(G20-G0)\times 0.9\times 100}{\mathrm{TS}}$$

(3) $$\mathrm{SDS}\;\left(\%\right)=\frac{(G120-G20)\times 0.9\times 100}{\mathrm{TS}}$$

(4) RS (%)=100−RDS−SDS

where Gt is glucose content at time t (mg); G20 and G120 are the glucose content released after starch digestion for 20 and 120 min, respectively; TS is the total starch content of the sample; and the factor conversion from glucose to starch is 0.9.

Digestion curves were fitted to a first-order kinetic model:

(5) $$\mathrm{Ct}=C\infty \times (1-e^{-\mathrm{kt}})$$

### 3.8. Analysis of Intermolecular Interaction Forces

The relative contributions of ionic bonds, hydrogen bonds, and hydrophobic interactions to the stability of PS–SJKP complexes were evaluated using specific chemical disruptors, according to the method of Yu et al. (2025) [35] with minor modifications. Freshly prepared PS–SJKP complexes were mixed with an equal volume of the following reagents: 0.5 M NaCl (to disrupt ionic bonds), 6 M urea (to disrupt hydrogen bonds), 2% (*w*/*v*) SDS (to disrupt hydrophobic interactions), and ultrapure water (control). The mixture was vortexed for 2 min and incubated at room temperature for 2 h. The final viscosity (FV) of each treated sample was measured using the RVA under the same conditions as described in Section 2.4.1. The relative change in FV was calculated as follows:

(6) $$\Delta \mathrm{FV}\;(\%)\;=\frac{{\mathrm{FV}}_{\mathrm{treated}}-{\mathrm{FV}}_{\mathrm{control}}\;}{{\mathrm{FV}}_{\mathrm{control}}\;}\times 100$$

A significant decrease in FV indicated the dominance of the corresponding molecular interaction in complex stabilization.

### 3.9. Antioxidant Activity Assays

The DPPH radical scavenging rate, ABTS radical scavenging rate, superoxide anion (O_2•_^−^) scavenging rate, and hydroxyl radical (•OH) scavenging rate of the samples were measured according to the method of Li et al. (2023) [36]. Freshly prepared PS–SJKP pastes were centrifuged at 8000× *g* for 10 min, and the supernatant was collected for antioxidant activity determination. Pure SJKP solutions with the same concentrations as the complexes were prepared as positive controls. All measurements were performed in triplicate, and the results were expressed as percentage scavenging activity.

### 3.10. Statistical Analysis

All experiments were performed in at least triplicate, and the data were expressed as mean ± standard deviation (SD). Statistical analysis was performed using SPSS 26.0 software (IBM Inc., Armonk, NY, USA). One-way analysis of variance (ANOVA) followed by Duncan’s multiple range test was applied to determine significant differences between groups (*p* ≤ 0.05). Pearson correlation analysis was performed to evaluate the relationship between structural parameters and functional properties. Curve fitting and graphing were conducted using Origin 2021 software (OriginLab Inc., Northampton, MA, USA).

## 4. Conclusions

This study revealed that sour jujube kernel peptide (SJKP) modulates pea starch digestibility in a nonlinear, concentration-dependent manner. At low SJKP (≤7.5%), hydrogen bonding enhanced short-range molecular order, structural compactness, and thermal stability, significantly reducing RDS and increasing RS. At high SJKP (≥10%), phase separation driven by hydrophobic interactions disrupted the structure, causing RDS to rebound to 58.30% and increasing antioxidant activity. The rheological and pasting properties further supported the concentration-dependent regulatory mechanism. At 7.5% SJKP, the complex exhibited the highest storage modulus (G′) and apparent viscosity, along with the lowest breakdown (BD) and setback (SB) values, indicating enhanced gel network stability and reduced retrogradation tendency. These changes are consistent with hydrogen bonding-dominated interactions at low SJKP concentrations, which strengthen the continuous gel matrix. In contrast, at high SJKP concentrations (≥10%), the marked decreases in G′ and viscosity, together with increased BD and SB, reflect the phase separation and structural collapse driven by hydrophobic interactions. Therefore, the pasting and rheological parameters not only confirm the structural transitions but also provide practical indicators for tailoring starch functionality in food processing.

These findings provide a revised mechanistic understanding of starch-peptide interactions based on hydrogen bonding and hydrophobic forces, offering a strategy for designing functional foods with controlled glucose release and antioxidant benefits. Considering the narrow diameter of amylose single helices (approx. 0.45–0.54 nm), it is sterically unlikely that bulky SJKP molecules are truly encapsulated; alternative mechanisms such as surface adsorption or network entrapment should be considered in future studies. Future research should focus on evaluating the long-term stability, sensory properties, and digestive behavior of the modified starch in real food systems.

## Figures and Tables

**Table 1:** Amino acid characteristics of sour jujube kernel peptide.

Amino Acid Name	Amino Acid Type	Amino Acid Content (mg/g)	Amino Acid Content (g/100 g Protein)	Amino Acid Reference Mode (mg/g Protein)	AAS (%)
**Essential amino acids (EAAs)**					
histidine	EAA, Basic	4.35	0.68	15.00	45.33
threonine	EAA, Hydrophilic	11.6	1.82	23.00	79.13
valine	EAA, BCAA, Hydroph	15.55	2.43	39.00	62.31
methionine + cysteine	EAA, S–containing	7.39	1.16	22.00	52.73
isoleucine	EAA, BCAA, Hydroph	8.24	1.29	30.00	43.00
leucine	EAA, BCAA, Hydroph	21.06	3.30	59.00	55.93
phenylalanine + tyrosine	EAA, AAA, Aromatic	18.95	2.97	38.00	78.16
tryptophan	EAA, Aromatic	0.24	0.04	6.00	6.67
lysine	EAA, Basic	22.70	3.55	45.00	78.89
**Nonessential amino acids (NEAAs)**					
aspartic acid	NEAA, Acidic	33.22	5.20	–	–
glutamate	NEAA, Acidic	60.56	9.48	–	–
asparagine	NEAA	0.30	0.05	–	–
serine	NEAA, Hydrophilic	24.41	3.82	–	–
glycine	NEAA	182.35	28.53	–	–
arginine	NEAA, Basic	50.37	7.88	–	–
alanine	NEAA, Hydrophobic	60.52	9.47	–	–
proline	NEAA, Hydrophobic	117.42	18.37	–	–
**Summary indicators**		Content (mg/g sample)	Content (g/100 g protein)		
Total amino acids (TAAs)	–	639.23	100.00	–	–
Total essential amino acids (TEAAs)	EAA	110.08	17.22	–	–
Branched-chain amino acids (BCAAs)	Ile, Leu, Val	44.85	7.02	–	–
Aromatic amino acids (AAAs)	Phe, Tyr, Trp	19.19	3.00	–	–
**Nutritional evaluation indicators**					
The first limiting amino acid	Tryptophan (AAS = 6.67%)				

**Table 2:** Pasting properties of different samples.

Polypeptide Addition	PT (°C)	PV (cp)	Pt (min)	TV (cp)	FV (cp)	BD (cp)	SB (cp)
0.0%	77.99 ± 0.57 ^b^	4001.84 ± 30.18 ^a^	4.71 ± 0.12 ^c^	1854.6 ± 22.97 ^c^	3251.28 ± 6.37 ^a^	2111.40 ± 9.87 ^a^	1388.60 ± 4.57 ^a^
2.5%	84.54 ± 1.19 ^a^	3584.72 ± 14.80 ^b^	5.08 ± 0.08 ^ab^	1982.12 ± 12.9 ^a^	2995.76 ± 9.53 ^b^	1580.68 ± 10.14 ^b^	1226.76 ± 4.68 ^b^
5.0%	84.06 ± 1.21 ^a^	3255.88 ± 22.29 ^c^	5.07 ± 0.04 ^b^	1904.8 ± 18.50 ^b^	3006.96 ± 5.52 ^b^	1366.40 ± 20.40 ^c^	1128.68 ± 2.16 ^c^
7.5%	84.04 ± 0.79 ^a^	3025.52 ± 10.78 ^d^	5.07 ± 0.03 ^b^	1688.48 ± 22.46 ^e^	2903.36 ± 14.79 ^c^	1340.80 ± 13.90 ^c^	997.08 ± 6.46 ^e^
10.0%	84.84 ± 1.21 ^a^	2950.16 ± 6.58 ^e^	5.20 ± 0.05 ^a^	1749.32 ± 5.37 ^d^	2818.8 ± 11.09 ^d^	1212.52 ± 5.53 ^d^	1045.76 ± 19.64 ^d^
12.5%	84.15 ± 0.56 ^a^	2651.08 ± 15.38 ^f^	5.10 ± 0.05 ^ab^	1574.84 ± 22.59 ^f^	2603.6 ± 17.82 ^e^	1124.28 ± 6.91 ^e^	915.92 ± 13.60 ^f^
15.0%	84.98 ± 0.66 ^a^	2550.72 ± 17.66 ^g^	5.22 ± 0.04 ^a^	1641.20 ± 22.10 ^e^	2440.12 ± 21.83 ^f^	902.00 ± 14.67 ^f^	912.24 ± 8.76 ^f^

Mean values from three repetitions ± SD. Values in columns not sharing the same letter are significantly different (*p* < 0.05).

**Table 3:** Thermal properties of different samples.

Polypeptide Addition	To (°C)	Tp (°C)	Tc (°C)	ΔH (J/g)	Tc–To (°C)
0.0%	61.33 ± 1.25 ^a^	66.55 ± 0.52 ^a^	73.82 ± 0.99 ^a^	10.85 ± 0.58 ^bc^	12.49 ± 1.59 ^ab^
2.5%	62.92 ± 0.82 ^b^	68.20 ± 0.54 ^b^	74.98 ± 0.75 ^b^	10.90 ± 0.63 ^bc^	12.06 ± 1.11 ^a^
5.0%	63.78 ± 0.77 ^bc^	69.00 ± 0.61 ^c^	76.08 ± 0.15 ^c^	11.45 ± 0.35 ^cd^	12.30 ± 0.78 ^ab^
7.5%	64.90 ± 0.64 ^cd^	70.30 ± 0.26 ^d^	76.46 ± 0.66 ^c^	11.73 ± 0.61 ^d^	11.56 ± 0.92 ^a^
10.0%	65.58 ± 0.53 ^de^	71.02 ± 0.13 ^e^	77.64 ± 0.46 ^d^	11.39 ± 0.27 ^cd^	12.06 ± 0.70 ^a^
12.5%	65.98 ± 0.63 ^e^	72.83 ± 0.99 ^f^	78.41 ± 0.35 ^e^	9.90 ± 0.66 ^a^	12.43 ± 0.72 ^ab^
15.0%	66.95 ± 0.19 ^f^	73.20 ± 1.10 ^f^	80.08 ± 1.08 ^f^	8.65 ± 0.22 ^a^	13.13 ± 1.10 ^b^

Mean values from three repetitions ± SD. Values in columns not sharing the same letter are significantly different (*p* < 0.05).

**Table 4:** The primary intermolecular forces in samples.

Polypeptide Addition	ΔFV (0.5 M NaCl)	ΔFV (6 M Urea)	ΔFV (2% SDS)	Dominant Force
0.0%	–2.10 ± 0.50 ^a^	–25.50 ± 1.80 ^d^	–5.50 ± 0.60 ^a^	Hydrogen bond
2.5%	+3.50 ± 0.70 ^b^	–38.70 ± 2.10 ^c^	–8.30 ± 0.90 ^ab^	Hydrogen bond
5.0%	–1.50 ± 0.40 ^a^	–45.20 ± 2.20 ^c^	–12.50 ± 1.00 ^b^	Hydrogen bond
7.5%	–1.80 ± 0.40 ^a^	–52.30 ± 2.50 ^e^	–10.20 ± 1.10 ^b^	Hydrogen bond
10.0%	+5.20 ± 0.90 ^b^	–35.10 ± 2.00 ^c^	–35.80 ± 1.80 ^c^	Hydrogen bond/hydrophobic
12.5%	–7.50 ± 0.80 ^c^	–26.80 ± 1.90 ^d^	–42.50 ± 2.00 ^d^	hydrophobic
15.0%	–4.30 ± 0.60 ^ab^	–18.50 ± 1.50 ^b^	–48.90 ± 2.20 ^e^	hydrophobic

FV: final viscosity measured by RVA (centipoise, cP). Mean values from three repetitions ± SD. Values in columns not sharing the same letter are significantly different (*p* < 0.05).

## Data Availability

The original contributions presented in this study are included in the article. Further inquiries can be directed to the corresponding author.

## Supplementary Materials

The following supporting information can be downloaded at: https://www.mdpi.com/article/10.3390/molecules31101718/s1. Table S1: Short-range ordered structure parameters (R1047/1022 and R995/1022) of PS-SJKP complexes.

## Abbreviations

The following abbreviations are used in this manuscript:

PS	Pea Starch
SJKP	Sour Jujube Kernel Peptide
SEM	Scanning Electron Microscope
XDR	X-ray Diffraction
FTIR	Fourier-transform Infrared Spectroscopy
RDS	Rapidly Digestible Starch
RS	Resistant Starch
SDS	Slowly Digestible Starch
LGI	Low-Glycemic Index
RVA	Rapid Visco Analyzer
DSC	Differential Scanning Calorimetry
