# Supplementary material for: The Interaction Between Sour Jujube Kernel Peptide and Pea Starch and Its Effects on Starch Properties and In Vitro Digestibility
Source: Molecules. 2026 May 19;31(10):1718. doi: 10.3390/molecules31101718 (PMC13210064; doi:10.3390/molecules31101718)
Supplement: Supplementary file 1 [file molecules-31-01718-s001.zip › molecules-4266888-supplementary.pdf]

Table S1. Short-range ordered structure parameters (R1047/1022 and R995/1022) of PS-SJKP complexes

SJKP concentration (%)	R1047/1022	R995/1022
0	0.682 ± 0.015^{d}	0.612 ± 0.022^{d}
2.5	0.745 ± 0.018^{c}	0.668 ± 0.035^{c}
5	0.821 ± 0.020^{b}	0.735 ± 0.017^{b}
7.5	0.895 ± 0.021^{a}	0.812 ± 0.016^{a}
10	0.856 ± 0.009^{b}	0.778 ± 0.016^{b}
12.5	0.765 ± 0.017^{c}	0.685 ± 0.024^{c}
15	0.698 ± 0.011^{d}	0.625 ± 0.013^{d}

Different superscript letters (a–d) within the same column indicate significant differences ($p < 0.05$) by one-way ANOVA followed by Duncan's multiple range test.
